# Identification of proteins interacting with the mitochondrial small heat shock protein Hsp22 of *Drosophila melanogaster*: Implication in mitochondrial homeostasis

**DOI:** 10.1371/journal.pone.0193771

**Published:** 2018-03-06

**Authors:** Afrooz Dabbaghizadeh, Geneviève Morrow, Yasmine Ould Amer, Etienne Hebert Chatelain, Nicolas Pichaud, Robert M. Tanguay

**Affiliations:** 1 Laboratoire de Génétique Cellulaire et Développementale, IBIS and PROTEO, Département de Biologie Moléculaire, Biochimie Médicale et Pathologie, Faculté de Médecine, Université Laval, Québec, Canada; 2 Laboratoire de Signalisation Mitochondriale, Département de Biologie, Université de Moncton, Moncton, NB, Canada; 3 Laboratoire de Biochimie et Physiologie Comparée, Département de Chimie et Biochimie, Université de Moncton, Moncton, NB, Canada; Universitair Medisch Centrum Groningen, NETHERLANDS

## Abstract

The small heat shock protein (sHsp) Hsp22 from *Drosophila melanogaster* (DmHsp22) is part of the family of sHsps in this diptera. This sHsp is characterized by its presence in the mitochondrial matrix as well as by its preferential expression during ageing. Although DmHsp22 has been demonstrated to be an efficient *in vitro* chaperone, its function within mitochondria *in vivo* remains largely unknown. Thus, determining its protein-interaction network (interactome) in the mitochondrial matrix would help to shed light on its function(s). In the present study we combined immunoaffinity conjugation (IAC) with mass spectroscopy analysis of mitochondria from HeLa cells transfected with DmHsp22 in non-heat shock condition and after heat shock (HS). 60 common DmHsp22-binding mitochondrial partners were detected in two independent IACs. Immunoblotting was used to validate interaction between DmHsp22 and two members of the mitochondrial chaperone machinery; Hsp60 and Hsp70. Among the partners of DmHsp22, several ATP synthase subunits were found. Moreover, we showed that expression of DmHsp22 in transiently transfected HeLa cells increased maximal mitochondrial oxygen consumption capacity and ATP contents, providing a mechanistic link between DmHsp22 and mitochondrial functions.

## Introduction

Protein homeostasis (proteostasis) has been reported to be implicated in many human degenerative diseases [1–4]. A number of protective mechanisms against damaged proteins in cytoplasm and cellular organelles have been described. Among them, the heat shock response (HSR) leading to expression of molecular chaperones is the first line against proteotoxicity [1, 2, 5]. Disruption of these mechanisms can lead to the accumulation of damaged proteins which are hallmarks of several diseases such as Parkinson’s and Alzheimer’s, both sharing mis-proteostasis as a common characteristic [6]. The accumulation of such deleterious proteins is also associated with aging [7, 8]. Despite significant progress in the understanding of the underlying mechanisms involved in protein homeostasis, many gaps remain to be elucidated [9]. Cellular protection by the HSR family members including Hsp70 [10, 11], Hsp90 [12] and small Heat shock proteins (sHsps) family members has been reported to play a role in maintaining protein homeostasis [13, 14]. Thus mitochondrial members of Hsp families may play essential roles in protein homeostasis within this important organelle.

Converting biochemical energy into ATP via the oxidative phosphorylation (OXPHOS) process by mitochondria is essential for multiple cellular reactions. Mitochondria also regulate many other vital physiological processes such as apoptosis, calcium homeostasis, reactive oxygen species (ROS) production, as well as anabolic and catabolic processes [15–19]. The mitochondrial proteome can influence functional and structural changes of the electron transport system (ETS), as well as differential ROS production. High ROS concentrations can be harmful and cause oxidative stress, which is believed to be the proximate cause of aging [20–25]. As the main producers of ROS in the cell, mitochondria are therefore prone to be damaged by ROS. To prevent intramitochondrial damages, the organelle is in constant cross talk with the nucleus via cellular signalling to transcriptionally activate genes involved in the mitochondrial unfolding protein response (mtUPR) [15–17, 23, 26, 27]. Mitochondrial dysfunctions have been associated with many pathophysiological conditions, as well as with senescence [21, 28–30]. A Protein Quality Control (PQC) machinery within mitochondria may be involved in many of these pathological processes [18]. This response referred to as the mitochondrial Unfolded Protein Response (mtUPR,) includes antioxidant enzymes, chaperones and proteases involved in cellular responses to mitochondrial damages and is responsible for maintenance of mitochondrial integrity [15].

The small heat shock proteins (sHsps) are expressed in all kingdoms of life and characterized by their small molecular weight, ranging from 12–42 kDa [1, 31–35]. *Drosophila melanogaster* encodes 12 proteins with the characteristic of sHsps [36]. *Drosophila melanogaster* Hsp22 (DmHsp22) is presently the only reported small Hsp that is constitutively localized in the mitochondrial matrix.

In the present study we sought to decipher mitochondrial network of DmHsp22 under heat shock and non-heat shock conditions. DmHsp22 was found to interact with many mitochondrial proteins including those of the ETS and the tricarboxylic acid cycle. Heat shock did not have a significant impact on the nature of mitochondrial partners of DmHsp22 as observed after recovery. Consistent with a role of DmHsp22 in maintaining mitochondrial homeostasis, HeLa cells over-expressing DmHsp22 exhibited higher maximal mitochondrial oxygen consumption capacity and ATP content suggesting modulation of mitochondrial functions as a result of elevated DmHsp22 activity.

## Materials and methods

### Construction of expression vectors

Full-length cDNA of DmHsp22 was inserted into the eukaryotic expression vector pcDNA^TM^3.1 (+) (Thermo Fisher Scientific) at Hind III and Xho I cloning sites. Polymerase chain reaction (PCR) (TransGen Biotech) and Gibson assembly (New England Biolabs) were used to construct three recombinant vectors pcDNADmHsp22, pcDNADmHsp22Flag and pcDNAFlag using oligonucleotide primers (Invitrogen) as described in Table 1. Constructs were verified by DNA profile upon restriction enzyme digestion on agarose gel and by DNA sequencing (Genomic Analysis Platform, Institut de Biologie Intégrative et des Systèmes (IBIS), Université Laval, Québec, Canada).

**Table 1:** Forward and reverse primers applied to the constructs of pcDNAHsp22 wild type, pcDNAHsp22WT-Flag and pcDNA-Flag.

Gene Identity	Sense	Primer sequence (5’-3’)
pcDNAHsp22WT	Fwd^a^	GGCTAGCGTTTAAACTTAATGCGTTCCTTACCGATG
	Rev^b^	ACGGGCCCTCTAGACTTACTGACTGGCGGCTTTGTC
pcDNAHsp22WT-Flag First PCR	Fwd^a^	CCCGGATCGGGGTACATGCGTTCCTTACCGATG
	Rev^b^	**GTCATCGTCTTTGTAGTC**CTGACTGGCGGCTTTGTC
pcDNAHsp22WT-Flag Second PCR	Fwd^a^	GGCTAGCGTTTAAAATTAATGCGTTCCTTACCGATG
	Rev^b^	ACGGGCCCTCTAGA**CTTACTTGTCGTCATCGTC**
pcDNAFlag	Fwd^a^	GGCTAGCGTTTAAACTTAAGCTTGGTACCGAGCTCGGA
	Rev^b^	ACGGGCCCTCTAGACTTA**CTTGTCGTCATCGTCTTTGTAGTC**

^a^Forward primer

^b^Reverse primer

The flag tag sequence is represented in bold.

### Cell culture and transfection

HeLa cells derived from human cervix adenocarcinoma [37] (a gift of Dr Josée N.Lavoie, Laval University) were grown in Dulbecco’s modified Eagle’s medium (DMEM, Gibco, Life Technologies), supplemented with 10% (v/v) fetal bovine serum (FBS; HyClone, Thermo Fisher Scientific), 2% (v/v) penicillin/streptomycine (Life Technologies/Gibco) and 2% (v/v) glutamax™ (Life Technologies/Gibco) to a final concentration of 50 units/ml penicillin/streptomycin and 0.9 mg.mL^-1^ glutamax™ in 5% CO_2_ humidity atmosphere at 37°C.

Cells were seeded with 2.3×10^5^ cells/35-mm dishes into 2 mL final volume medium and transiently transfected at exponential growth phase with 3 μg of plasmids using 3 μL of Lipofectamine®2000 as transfection agent (Invitrogen). Cells were ~ 80–85% confluency at the day of transfection. Opti-MEM® (Life Technologies/Gibco) was used to the final volume of 500 μl into 35-mm dishes to improve effectiveness of transfection. Cells were harvested 24 hours post-transfection.

### Immunofluorescence microscopy

HeLa cells were seeded on glass coverslips and transfected with constructs cited above. After 24 hours, cells were fixed in 3.7% paraformaldehyde for 10 min and 0.1% Triton X-100 for 5 min. They were blocked in 5% Bovine Serum Albumin (BSA) (Hyclone) in PBS containing 0.2% Tween for 1 hour at room temperature (RT). Cells were exposed to primary antibody produced in rabbits by immunization using a bacterial DmHsp22 fusion protein in the pET3 vector (Novagen), diluted 1:1000 [38] and secondary antibody Fluorescein isothiocyanate conjugated donkey secondary antibodies against rabbit IgG (Invitrogen) diluted 1:500 in PBS-5% BSA for 1 hour at RT. 4,6-diamidino-2-phenylindole (DAPI) was used at the final concentration of 0.2 μg. mL^-1^ for 1 min to stain the nuclei. Coverslips were mounted in Vectashield mounting medium (Vector Laboratories). Images were acquired using an Axio Observer Z1 microscope equipped with an Axiocam camera (Carl Zeiss, Canada).

### Cellular fractionation

Transfected HeLa cells were washed, trypsinized and scraped using cell scraper (Greiner, Bio-one) into the media followed by centrifugation at 1000 g for 5 min at 4°C. Media was removed after centrifugation and RIPA (50 mM Tris, pH 8.0, 150 mM NaCl, 1% NP 40, 1 mM PMSF) was added to the pellet according to the number of harvested cells (~1.2x10^6^ cells in 150 μL). The lysate was kept on ice for 10 minutes and centrifuged at 1300 g for 5 minutes at 4°C. Supernatant (S1300) containing the proteins were collected for further analysis, including western blot (WB) and immune affinity capture (IAC). To extract mitochondria, the cell pellet was re-suspended in ice-cold RSB hypo buffer (10 mM NaCl, 1.5 mM MgCl2, 10 mM Tris-HCl pH 7.5) and transferred to a Dounce homogenizer [39]. Cell suspension was kept on ice for 10 min to swell, and swollen cells were mechanically broken using Teflon Dounce homogenizer while keeping them on ice. Several passages through sterile 28 G needle (PrecisionGlide® Needle) were performed to optimize degree of cell disruption. Immediately, mannitol and sucrose were added to final concentrations of 210 mM and 70 mM, respectively. Cells were re-centrifuged at 1300g for 5 min at 4°C. The supernatant was transferred into a new centrifuge tube and mitochondria were isolated by centrifugation at 12,000 g for 15 min at 4°C. Mitochondrial pellet was washed twice with MS homogenization buffer 1× (210 mM mannitol, 70 mM sucrose, 5 mM Tris-HCl, 1mM EDTA pH 7.5) [39].

### Electrophoresis and western blot analysis

Proteins were separated on SDS-polyacrylamide gel electrophoresis (PAGE) [40], transferred onto nitrocellulose membranes (BioTrace™ NT) and membranes were blocked in 5% dry milk containing 0.1–0.2% Tween-20 (TBST). Western blots were performed with the primary antibodies: anti-Hsp22 (number 36, 1:5000) produced in rabbits as mentioned above [38], anti-Hsp60 (number 37, 1:10,000) [41], anti-cytochrome c (1:250, Pharmingen), anti-Hsp90 (1:5000) and anti-mtHsp70 (diluted 1:5000) [42]. Peroxidase-labeled secondary antibodies were used (1:5000, Jackson Immuno Research Laboratories). Peroxidase activity on membrane was visualized on a LI-COR imager using chemiluminescent reagent (BioRAD). Molecular weight markers were used to estimate the size of the proteins between 10–250 kDa (Bio-Rad).

### Immuno affinity capture experiments

1–2 mg of S1300 containing the proteins after Bradford protein assay were moved into clean Eppendorf tubes. 100–200 μl of washed anti-DYKDDDDK (Flag) (Biotool) affinity gel suspension was rinsed three times by using Tris-buffered saline (TBS) (1×) (50 mM Tris-HCl, 150 mM NaCl, pH 7.5) plus 0.05% Tween-20 (Fisher Scientific) and was added to each tube immediately after centrifugation under end-to-end rotation for two hours at 4°C. At the end of the incubation, suspension was centrifuged for 30 seconds at 5000g at 4°C and the supernatant was identified as the unbound fraction. To remove the detergent and unspecific binders, beads were washed 3–4 times with 500 μl of TBS. Finally, beads were washed 4–5 more times with ammonium bicarbonate 50 mM prior sending to Proteomics Platform of the CHU de Quebec Research Center, Quebec, Canada for further analysis.

### Mass spectrometry analysis

Proteins on beads were suspended in 25 μl of 50 mM ammonium bicarbonate, 1 μg trypsin was added and sample was incubated overnight at 37°C. Trypsin reaction was stopped by acidification with 3% acetonitrile-1% TFA-0.5% acetic acid. Beads were removed by centrifugation, the peptides were purified from supernatant on stage tip (C18) and vacuum dried before MS injection. Sample is solubilized into 10 μl of 0.1% formic acid and 5 μl were analyzed by mass spectrometry. Peptide samples were injected and separated by online reversed-phase (RP) nanoscale capillary liquid chromatography and analyzed by electrospray mass spectrometry (ESI MS/MS). The experiments were performed with a Dionex UltiMate 3000 nanoRSLC chromatography system (Thermo Fisher Scientific / Dionex Softron GmbH, Germering, Germany) connected to an Orbitrap Fusion mass spectrometer (Thermo Fisher Scientific, San Jose, CA, USA) using Orbitrap Fusion Tune Application 2.0 and equipped with a nanoelectrospray ion source.

Peptides were trapped at 20 μL.min^-1^ in loading solvent (2% acetonitrile, 0.05% TFA) on a 5 mm x 300 μm C18 pepmap cartridge pre-column (Thermo Fisher Scientific / Dionex Softron GmbH, Germering, Germany) during 5 minutes. Mass spectra were acquired using a data-dependent acquisition mode using Thermo Xcalibur software (version 3.0.63). The research was performed on Uniprot *Homo sapiens* corresponding protein interacting DmHsp22-Flag and pcDNA-Flag. Scaffold (version Scaffold_4.8.4, Proteome Software Inc., Portland, OR) was used to validate MS/MS based peptide and protein identifications. Peptide identifications were accepted if they could be established at greater than 33.0% probability to achieve an FDR (false discovering rate) less than 1.0% by the Scaffold Local FDR algorithm. Protein identifications were accepted if they could be established at greater than 99.0% probability to achieve an FDR less than 1.0% and contained at least 3 identified peptides. Protein probabilities were assigned by the Protein Prophet algorithm [43]. Quantitative analysis was performed using emPAI (exponential modified protein abundance index) to estimate protein content in a matrix [44].

### Mitochondrial oxygen consumption

HeLa cells were trypsinized, washed and suspended at a final concentration of 1×10^6^ cells. mL^-1^ in serum free DMEM. Briefly, mitochondrial respiration was measured [45] using an Oxygraph O2k (Oroboros company, Innsbruck, Austria), in 2 mL glass chambers equipped with polarographic electrodes, with 750 rpm stirring rate at 37°C calibrated to oxygen air-saturation in DMEM. Intact cells were used to measure endogenous respiration (N = 7). After transfer of the cells to the oxygraph, the chambers were closed and the cellular ROUTINE respiration supported by endogenous substrates or exogenous substrates in the culture media (glucose) was measured. Oligomycin (2 μg. mL^-1^) was then used to inhibit the ATP synthase and determine the LEAK respiration, which corresponds to non-phosphorylating respiration, when oxygen consumption is maintained to compensate the proton leak. Carbonyl cyanide-p-trifluoro-methoxyphenylhydrazone (FCCP), a protonophore uncoupler, was then carefully titrated using steps of 0.5 μM, until reaching optimal concentration in order to achieve the maximal noncoupled respiration rate representing the respiration at maximal capacity of the ETS. Finally, Rotenone (0.5 μM) and Antimycin A (2.5 μM) were added to inhibit complexes I and III of the ETS respectively and measure the residual oxygen consumption, i.e. the oxygen consumption due to oxidation side-reactions. Mitochondrial oxygen consumption was expressed as pmol O_2_. s^-1^. 10^−6^ cells.

### ATP content measurements

ATP content of the cells was measured using the ATP Bioluminescence Assay Kit HSII (Roche), according to the manufacturer’s protocol. Briefly, 1–4×10^6^ cells were lysed with the cell lysis reagent and the amount of cellular ATP was measured and evaluated using a luciferase-based assay and Varioskan flash multimode microplate reader (Thermo Fisher Scientific). This assay uses firefly luciferase to convert ATP and luciferin to oxyluciferin and light. The light emitted in this reaction is directly proportional to the concentration of ATP. Two different conditions were tested: (i) without rotenone and antimycin inhibitors, allowing the measurement of total ATP content of the cell, and (ii) with rotenone and antimycin A (1 μM and 2.5 μM, respectively) to inhibit complexes I and III of the ETS and determine the ATP content related to glycolysis (N = 4 for each condition). Specific mitochondrial ATP levels were calculated as Total ATP content (i)–Glycolytic ATP content (ii).

### Luciferase reactivation assay

Cells transiently expressing Super Luciferase-eGFP containing 1–27 amino acids of DmHsp22 at its N terminus (for mitochondrial localization) (a kindly gift from Jurre Hageman, University of Groningen, Netherlands) were transfected with DmHsp22 and control vector. Cells were treated with cycloheximide (final concentration of 20 μg. mL^-1^ of growth media) 24 hours post-transfection to prevent *de novo* synthesis of proteins *in vivo* during the experiment. Cells in sealed plates were exposed to heat stress from 40–46°C in water bath for 30 or 60 minutes and cells were lysed in RIPA buffer following heat shock. Luciferin (Sigma) (0.4 mM) was added to the 50 μL of cell lysate at the same volume of refolding buffer (1.6 mM ATP, 5 mM MgSO_4_, 0.5 mM EDTA, 0.1 mg. mL^-1^ BSA in 25 mM Tricine, pH 7.8) and refolding was allowed to process by incubation at 37°C for 6 hours. Finally, luciferase activity was measured on a luminometer (LKB Wollac, Turku, Finland, model 1250) according to the standard protocol. Data are representative of four independent assays, and expressed as percentage of luciferase activity after 6 hours of recovery as means ± STD.

## Results

### Mitochondrial localization of DmHsp22-Flag in transiently transfected HeLa cells

**Fig 1:**
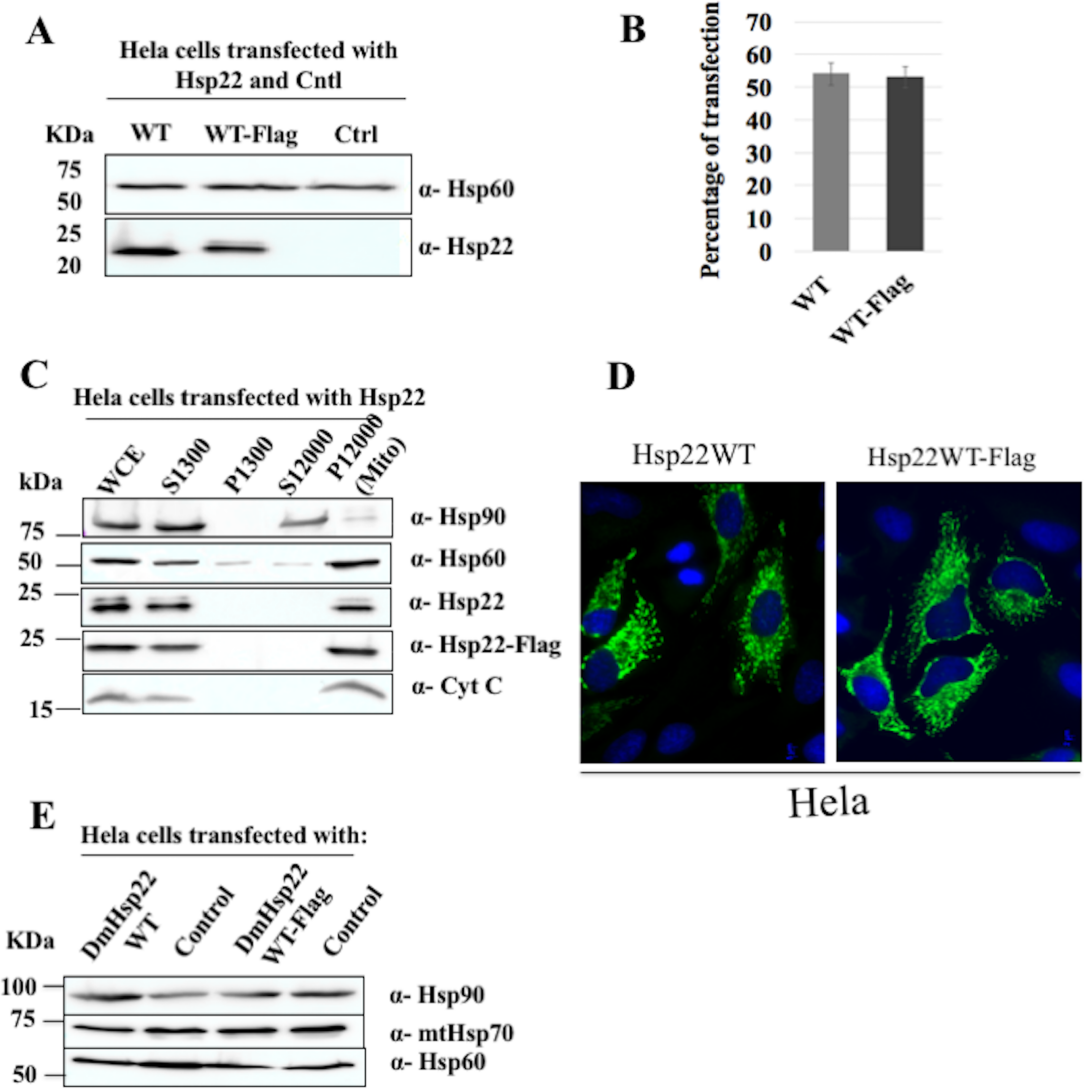
Characterization of DmHsp22WT and DmHsp22WT-Flag expressing cells. (A) HeLa cells were transfected with 3 μg of DmHsp22WT, WT-Flag and pcDNA as negative control (Ctrl) and 3 μl of lipofectamine as transfection reagent at 1:1 ratio for 24 hours. Cells were lysed with RIPA buffer and western blot was performed. DmHsp22’s expression level was detected in immunoblot probed with anti-DmHsp22 antibody. Anti-Hsp60 was used as loading control (see Materials and Methods). (B) Percentage of transfected cells (% of total cells) was determined 24 hours after transfection using immunofluorescence. Results are shown as a bar graph (P>0.05). Values represent the means ± SD from ten independent experiments. (C) Whole-cell extracts (WCE), cytosolic extracts (S1300 or S12000g), pellets (P1300g) and purified mitochondria fractions (P12000 or Mito) from HeLa cells transfected with DmHsp22WT and WT-Flag. 25 μg of each fraction were analysed by SDS-Page followed by immunoblotting with Anti-Hsp60 and Anti-cytochrome c as mitochondrial markers and anti-Hsp90 as a cytosolic marker. (D) HeLa cells expressing DmHsp22WT and C-terminal Flag-tagged DmHsp22 were processed for immunofluorescence detection of DmHsp22 as described in Materials and Methods. 24 hours post-transfection, fixed and permeabilized cells were stained with anti-Hsp22 antibody followed by Cy2 (green fluorescence) and DAPI (blue fluorescence) for nuclei labelling. Images were obtained using a fluorescence microscope. (E) Expression of DmHsp22WT and WT-Flag did not change levels of Hsp60 and Hsp70 in immunoblots probed with the corresponding antibodies. Anti-Hsp90 was used as loading control.

DmHsp22WT and DmHsp22WT-Flag were transfected in HeLa cells and their expression was confirmed using immunoblot on whole-cell extracts with mitochondrial Hsp60 as a mitochondrial marker (Fig 1A). Transfection efficiency of DmHsp22WT and DmHsp22WT-Flag was evaluated at ~ 55% by fluorescence microscopy (Fig 1B). The mitochondrial localization of DmHsp22 constructs was confirmed in a 12,000g mitochondrial pellet (Fig 1C). This indicates that the mitochondrial-targeting signals involved in their localization are recognized in mammalian cells. Immunofluorescence microscopy results confirm that both constructs are localized in mitochondria, and the presence of the tag has no obvious effect on its localization (Fig 1D). Moreover, the expression of DmHsp22 or its Flag version did not influence the level of expression of human mitochondrial chaperones Hsp60 and mtHsp70 (Fig 1E) suggesting that the exogenous expression did not cause a significant stress for the cells.

### Identification and characterization of DmHsp22-associated proteins

**Fig 2:**
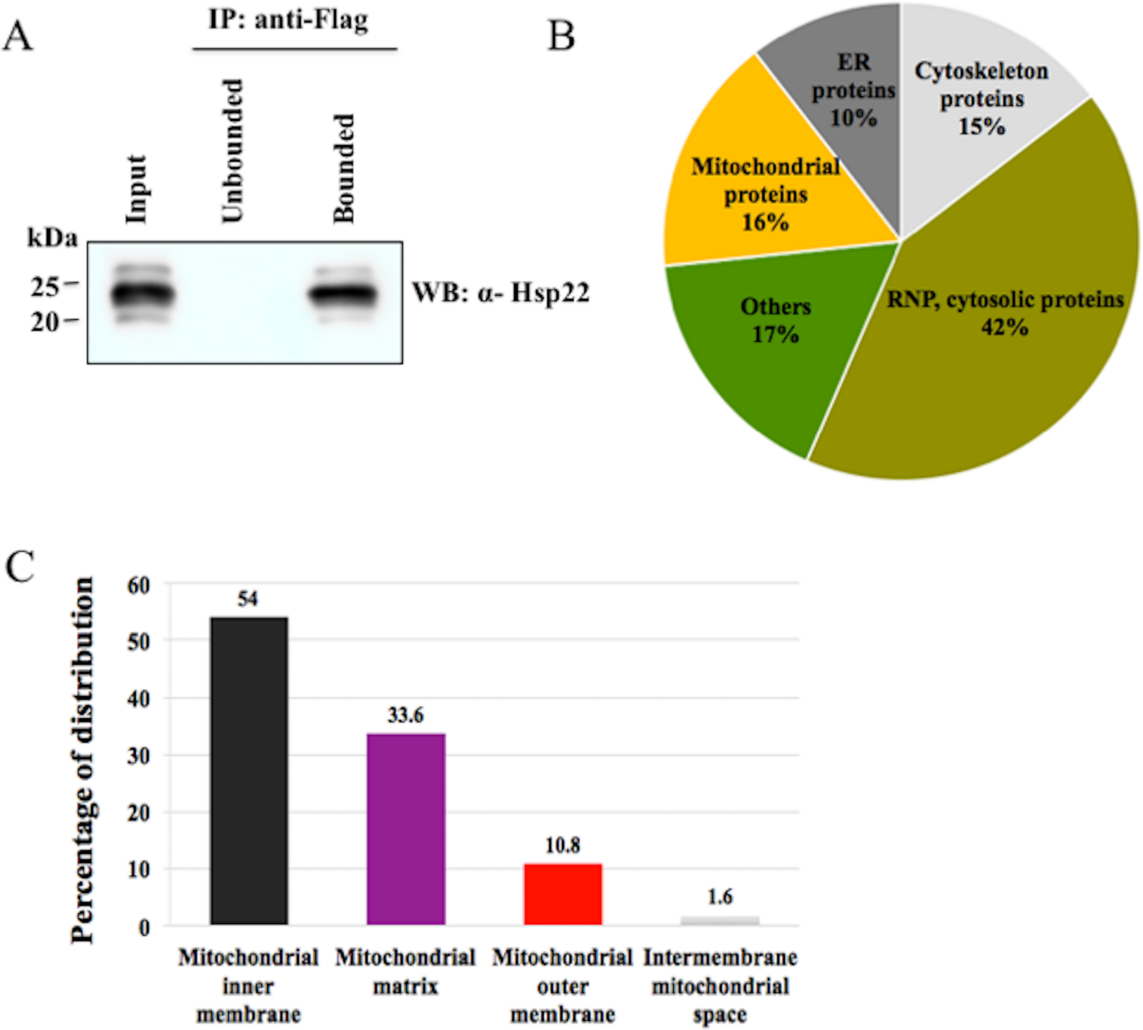
Identification of DmHsp22-associated proteins. (A) Immunoaffinity capture was performed using Anti-Flag monoclonal antibody that covalently attached to sepharose beads. Input lane contains S1300 (Supernatant of 1300 g). Input (S1300) was incubated for 2 hours at 4°C followed by centrifugation to separate unbounded and bounded fractions to the beads. Both experiments were performed with 2 and 1 mg protein from S1300 extracts of HeLa cells expressing DmHsp22 and control vector. The presence of DmHsp22 in each fraction was analysed with immunoblotting using a polyclonal antibody against DmHsp22 (N = 3). (B) Organelle distribution of DmHsp22-associated proteins (%) identified by the orbitrap fusion LC-MS/MS mass spectrometer. Analysis was obtained according to the Uniprot identifiers information. (C) Distribution of detected proteins in different sub-compartments of mitochondria were identified by PANTHER online tool.

Immuno-affinity conjugation was used to identify the mitochondrial DmHsp22-associated proteins first on a 1300 g supernatant (S1300) of transfected HeLa cells. pcDNA empty vector was used as a negative control. Two independent IAC experiments were performed on the S1300 fraction and the efficiency of binding is shown in Fig 2A. To identify the proteins that specifically interact with DmHsp22, we used mass spectrometry. The proteins that show a significant differential emPAI abundance index between the samples and the control are shown in Table 2.

**Table 2:** Proteins interacting with DmHsp22 by orbitrap fusion LC-MS/MS mass spectrometer.

**Detected proteins**	**IAC on S1300 of HeLa cells**					
**Detected proteins (Accession number)**	**First IAC**			**Second IAC**		
	**DmHsp22-Flag**		**Control**	**DmHsp22-Flag**	**Control**	**Heat Shock +6 h post-recovery**
ADP/ATP translocase2, SLC25A5 (P05141)	27.5		3.33	9.13	1	4
ADP/ATP translocase3, SLC25A6 (P12236)	16.2		1.5	7.26	0.78	3.18
Isoform 2 of ATPase family AAA domain-containing protein 3A (Q9NV17-2)	12.4		0.6	4.81	1	2
ATPase family AAA domain-containing protein 3B (Q5T9A4)	5.11		0	0	0	0
**ATP synthase subunit alpha, mitochondrial (P25705)**	**7.2**		**2.2**	**21.5**	**1.5**	**12**
**ATP synthase subunit beta, mitochondrial (P06576)**	**10.6**		**3.5**	**10**	**2.99**	**6.53**
**ATP synthase subunit g, mitochondrial (E9PN17)**	**10.4**		**0.2**	**7**	**1**	**6**
**ATP synthase subunit gamma (P36542)**	**5.5**		**0.5**	**2.5**	**0.14**	**1.3**
**Isoform Heart of ATP synthase subunit gamma, mitochondrial (P36542)**	**4.82**		**0.5**	**2.5**	**0.1**	**2**
**ATP synthase subunit e, mitochondrial (P56385)**	**4.51**		**0**	**8.35**	**3.6**	**6**
**ATP synthase subunit d, mitochondrial (O75947)**	**3.86**		**0.8**	**3.5**	**1.1**	**1.85**
**ATP synthase subunit epsilon (P56381)**	**3.65**		**0.4**	**0**	**0**	**0**
**ATP synthase subunit O, mitochondrial (P48047)**	**2.12**		**0.8**	**3.67**	**0.64**	**3.16**
**ATP synthase F(0) complex subunit B1, mitochondrial (P24539)**	**0.54**		**0**	**0**	**0**	**0**
**ATP synthase subunit delta, mitochondrial (P30049)**	**0.43**		**0**	**3.47**	**1.1**	**1.99**
Phosphate carrier protein, mitochondrial (F8VVM2)	7.6		0.6	4.48	0.32	1.8
Isoform 2 of Calcium-binding mitochondrial carrier protein Aralar1, SLC25A12 (F8W9J0)	2.2		0.12	0.91	0.12	0.84
Calcium-binding mitochondrial carrier protein SCaMC-1, SLC25A24 (Q6NUK1)	0.13		0	0.44	0	0.23
Calcium-binding mitochondrial carrier protein Aralar2, SLC25A13 (Q9UJS0)	3.3		0.15	1.7	0	1.12
Tricarboxylate transport protein, mitochondrial SLC25A1 (P53007)	1		0.2	0	0	0
Mitochondrial 2-oxoglutarate/malate carrier protein, SLC25A11 (Q02978)	1		0	1	0	1.11
Mitochondrial thiamine pyrophosphate carrier (Q9HC21)	0.43		0	0	0	0
Mitochondrial glutamate carrier 1, SCL25A22 (Q9D6M3)	0.35		0	0.72	0.14	0.86
Solute carrier family 25 member 46 (Q96AG3)	0.16		0	1.8	0	0.1
60 kDa heat shock protein, mitochondrial (P10809)	9		1	27.8	6	16.8
Stress-70 protein, mitochondrial (P38646)	2.65		0.7	1.32	0.8	1.23
HSP 90-beta (P08238)	2.58		1.25	2.87	0.9	0
Elongation factor Tu, mitochondrial (P49411)	4		1.27	19	7	16.7
78 kDa glucose-regulated protein (P11021)	8.74		1.5	5.5	2.8	0
Prohibitin (P35232)	4.18		1	1.4	1.7	0
Probibitin-2 (J3KPX7)	3		0.6	1	1	1.52
Glutamate dehydrogenase 1, mitochondrial, (P00367)	2.4		0	2.85	0.1	0.76
Voltage-dependent anion-selective channel protein 1 (P21796)	4		0	1.32	0.39	2.69
Voltage-dependent anion-selective channel protein 2 (A0A0A0MR02)	1.15		0.23	0	0	0
Mitochondrial import receptor subunit TOM22 (Q9NS69)	1.8		0.51	0	0	0
Mitochondrial import receptor subunit TOM70 (O94826)	0.1		0	0	0	0
Mitochondrial import inner membrane translocase subunit TIM50 (Q3ZCQ8)	0.84		0	1.78	0	1
Mitochondrial import inner membrane translocase subunit TIM14 (Q96DA6)	0.6		0	0	0	0
Mitochondrial import inner membrane translocase subunit TIM44 (O43615)	0.45		0	0	0	0
Mitochondrial import inner membrane translocase subunit TIM23 (O14925)	0.34		0	3	0	3
**Detected proteins**	**IAC on S1300 of HeLa cells**					
**Detected proteins (Accession number)**	**First IAC**			**Second IAC**		
	**DmHsp22-Flag**	**Control**		**DmHsp22-Flag**	**Control**	**Heat Shock +6 h recovery**
Putative mitochondrial import inner membrane translocase subunit Tim23B (Q5SRD1)	0	0		1.18	0	0.44
MICOS complex subunit MIC60 (B9A067)	1.55	0		0.78	0	0.25
MICOS complex subunit MIC13 (Q8R404)	1.31	0.1		4.73	0	2.57
MICOS complex subunit MIC27 (Q6UXV4)	0.25	0		0	0.14	0
MICOS complex subunit MIC19 (Q9NX63)	1.34	0.38		4.16	0	1.44
MICOS complex subunit MIC 25 (Q9BRQ6)	0	0		4.16	0	
Cytochrome b-c1 complex subunit 2, mitochondrial (P22695)	1	0		1.46	0.4	0.9
Cytochrome b-c1 complex subunit Rieske-like protein 1 (P47985)	0.24	0		0	0	
Cytochrome c oxidase subunit 6C (P09669)	0.88	0		1.39	0	0
Cytochrome c oxidase assembly factor 3 homolog, mitochondrial (Q9Y2R0)	1.5	0		0	0	
Cytochrome c oxidase subunit NDUFA4 (O00483)	0.83	0		0	0	0
Cytochrome c oxidase subunit 2 (P00403)	0.62	0		0	0	0
Cytochrome c1, heme protein, mitochondrial (P08574)	0.43	0.19		0.67	0	0.48
Cytochrome c oxidase subunit 4 isoform 1, mitochondrial (P13073)	0.38	0		1.4	0	0
Ubiquinol-cytochrome-c reductase complex assembly factor 3 (Q6UW78)	0.76	0		0	0	
NADH-cytochrome b5 reductase 3 (P00387)	0.45	0		0	0	
NADPH-cytochrome P450 reductase (E7EMD0)	0.15	0.04		0.15	0	
NADH dehydrogenase 1 alpha subcomplex subunit 9, mitochondrial (Q16795)	1	0.16		0	0	
NADH dehydrogenase 1 beta sub-complex subunit 10 (H3BPJ9)	0.84	0.11		0	0	0
NADH dehydrogenase 1 beta sub-complex subunit 6, mitochondrial (O95139)	0.5	0.14		0	0	
NADH dehydrogenase iron-sulfur protein 8, mitochondrial (E9PN51)	0.61	0		0	0	0
NADH dehydrogenase iron-sulfur protein 3, mitochondrial (O75489)	0.51	0		0	0	
NADH dehydrogenase 1 beta subcomplex subunit 5, mitochondrial (E7EWP0)	0.37	0.1		0	0	
Succinate dehydrogenase flavoprotein subunit, mitochondrial (D6RFM5)	0.36	0.14		0.78	0.23	0.25
NADH dehydrogenase iron-sulfur protein 7, mitochondrial (A0A087WT13)	0.31	0.09		0	0	
NADH dehydrogenase 1 alpha subcomplex subunit 5 (A0A087X1G1)	2.51	0		0	0	
NADH-ubiquinone oxidoreductase 75 kDa, mitochondrial (P28331)	0.1	0		0	0	
NAD(P) transhydrogenase, mitochondrial (Q13423)	0.26	0		0.19	0	0.09
NADH dehydrogenase iron-sulfur protein 2, mitochondrial (O75306)	0.13	0		0	0	
Acetyl-CoA acetyltransferase (P24752)	0.33	0.1		0	0	
Up-regulated during skeletal muscle growth protein 5 (Q96IX5)	6.14	0.35		0	0	
Carbamoyl-phosphate synthase (ammonia), mitochondrial (P31327)	0.7	0.6		16	2	
KN motif and ankyrin repeat domain-containing protein 2 (Q63ZY3)	3	0		1.6	0	
Sideroflexin (A0A0A0MS41)	1.93	0		4.4	0.3	2.78
Sideroflexin-1 (Q9H9B4)	0.68	0.12		2	0.2	1
Trifunctional enzyme subunit alpha, mitochondrial (P409339)	0.47	0.16		0.06	0	0.04
Trifunctional enzyme subunit beta (B5MD38)	0.88	0.25		0	0	0
Stomatin-like protein 2, mitochondrial (Q9UJZ1)	1.13	0.11		0.51	0.4	
Sulfide:quinone oxidoreductase, mitochondrial (Q9Y6N5)	1	0		0.88	0	
**Detected proteins**	**IAC on S1300 of HeLa cells**					
**Detected proteins (Accession number)**	**First IAC**			**Second IAC**		
	**DmHsp22-Flag**		**Control**	**DmHsp22-Flag**	**Control**	**Heat Shock +6 h recovery**
Aspartate-tRNA ligase, mitochondrial (Q6PI48)	1		0	0.92	0	
Serine/threonine-protein phosphatase PGAM5, mitochondrial (Q96HS1)	1		0	0	0	
Isoform 2 of Carnitine O-palmitoyltransferase1, liver isoform (P50416)	0.86		0	0.25	0	0.08
Isoform 3 of pyruvate dehydrogenase E1 component subunit alpha, mitochondrial (P08559)	0.54		0	0.9	0.33	0.67
Glutaryl-CoA dehydrogenase, mitochondrial (Q929470)	0.67		0	0	0	
Pyruvate dehydrogenase E1 component subunit beta, mitochondrial (P11177)	0.61		0.17	1	0.2	0.1
DNA-3-methyladenine glycosylase (P29372)	0.47		0.06	0	0	
Phosphatidyl glycerophosphatase and protein-tyrosine phosphatase1 (Q8WUK0)	0.33		0.1	0	0	
Methylcrotonyl-CoA carboxylase subunit alpha, mitochondrial (Q96RQ3)	0		0	0.34	0	
Isoform 3 of Carbamoyl-phosphate synthase, mitochondrial (P31327)	0		0	0.74	0.04	
Pyruvate carboxylase, mitochondrial (P11498)	0		0.04	1	0.1	0.06
Hydroxysteroid dehydrogenase-like protein 2 (Q6YN16)	0.16		0	0	0	
Succinyl-CoA ligase subunit beta, mitochondrial (Q96199)	0.16		0	0	0	
Saccharopine dehydrogenase-like oxidoreductase (Q8NBX0)	0.15		0	0	0	
Probable arginine-tRNA ligase (Q5T160)	0.11		0	0	0	
Lysine-tRNA ligase (Q15046)	0.1		0	0	0	
Valine-tRAN ligase (P26640)	0.1		0.06	0.11	0.1	0.1
DnaJ homolog subfamily A member 1 (P31689)	2		0.34	0	0	
Erythrocyte band 7 integral membrane protein (P27105)	1.43		0.31	2.27	0.5	
Arginine and glutamate-rich protein 1 (Q9NWB6)	1.36		0.4	0.72	0.76	
ATP-binding cassette sub-family F member 2, mitochondrial (Q9UG63)	0.32		0.02	1.95	0.33	0.9
ATP-dependent Clp protease ATP-binding subunit clip-like, mitochondrial (O76031)	1		0	0	0	
Putative coiled-coil-helix-coiled-coil domain-containing protein (Q9BUK0)	0.5		0	5	2	4
Evolutionary conserved-signalin intermediate in Toll pathway, mitochondrial (K7EJG5)	1		0	0	0	
Protein MTO1 homolog, mitochondrial (Q9Y2Z2)	0.91		0	0	0	
Protein NipSnap homolog 1 (Q9BPW8)	0.73		0	0.32	0.1	0.37
60S ribosomal protein L35a (P18077)	0.6		0.6	7	5.2	
Isoform 2 of HCLS1-associated protein X-1 (O00165)	0.5		0	0.53	0.38	0.38
Isoform 4 of Clusterin (P10909-4)	0.5		0	0	0	
AarF domain-containing protein kinase 4 (M0QZZ2)	0.47		0.13	0	0	
V-type ATPase catalytic subunit A (P38606)	0.45		0.06	0	0	
39S ribosomal L13, mitochondrial (Q9BYD1)	0.44		0	0	0	
Mitochondrial inner membrane protein OXA1L (Q15070)	0.42		0	0	0	
GTPase Era, mitochondrial (O75616)	0.31		0.04	0	0	
Annexin A1 (P04083)	0.4		0	0	0	
Peptidyl-Trna hydrolase 2, mitochondrial (Q9Y3E5)	0.38		0.11	0	0	
Complement component 1 Q subcomponent-binding protein, mitochondrial (Q07021)	0.37		0	0	0	
Apoptosis-inducing factor 1, mitochondrial (O95831)	0.34		0	1.2	0.1	0.44
Aldehyde dehydrogenase X, mitochondrial (P30837)	0.263		0.07	0.28	0	0.13
**Detected proteins**	**IAC on S1300 of HeLa cells**					
**Detected proteins (Accession number)**	**First IAC**			**Second IAC**		
	**DmHsp22-Flag**	**Control**		**DmHsp22-Flag**	**Control**	**Heat Shock +6 h recovery**
Dnaj homolog subfamily A member 3, mitochondrial (Q96EY1) (Q96EY1)	0.3	0		0	0	0
Danj homolog subfamily C member 11 (Q9NVH1)	0.237	0		0	0	0
Alkyldihydroxyacetonephosphate synthase (O00116)	0.2	0		0	0	0
Succinyl-CoA ligase subunit alpha, mitochondrial (P53597)	0.2	0.12		0	0	0
Protein-glutamine gamma-glutamyltransferase 2 (P21980)	0.2	0.02		0.2	0.3	
Putative ribosome-binding factor A, mitochondrial (Q8N0V3)	0.2	0.05		0	0	
NADH dehydrogenase flavoprotein 1, mitochondrial (B4DE93)	0.18	0		0	0	
Dimethyladenosine transferase 1, mitochondrial (Q8WVM0)	0.2	0.05		0	0	
Long-chain-fatty-acid-CoA ligase 3 (O95573)	0.185	0.02		1.18	0.1	0.19
Synaptic vesicle membrane protein VAT-1 homolog (Q99536)	0.17	0		0	0	
Glutamine-tRNA ligase (P41250)	0.08	0.02		0	0	
ATP-binding cassette sub-family B member 6, mitochondrial (Q9NP58)	0.07	0		0	0	
2-oxoglutarate dehydrogenase, mitochondrial (A0A0D9SFS3)	0.06	0		0	0	
Activating molecule in BECN1-regulated autophagy protein 1 (Q9C0C7)	0.05	0.01		0	0	
Putative ATP-dependent RNA helicase DHX30 (H7BXY3)	0.05	0.03		2	3	
Microtubule-associated tumor suppressor 1 (Q9ULD2)	0.05	0		0.28	0	
Serine/threonine-protein kinase mTOR, mitochondrial (P42345)	0	0		0.07	0	
Single-stranded DNA-binding protein, mitochondrial (Q04837)	0	0		1.12	0.48	
AFG3-like protein 2 (Q9Y4W6)	0	0		0.6	0	
Isoform 2 of Probable hydrolase PNKD (Q8N490)	0	0		1.9	0.14	
Dihydrolipoyl dehydrogenase (E9PEX6)	0	0		0.43	0	
Feline leukemia virus subgroup C receptor-related protein 1 (Q9Y5Y0)	0.05	0		0.48	0.26	
Clathrin heavy chain (A0A087WVQ6)	0.02	0		0.41	0.25	
Staphylococcal nuclease domain-containing protein 1(Q7K2F4)	0	0		0.8	1.2	
Peptide-prolyl cic-trans isomerase FKBP8 (A0A0A0MTJ1)	0	0		1.23	0.28	
Isoform 3 of cold shock domain-containing protein E1 (O75734)	0	0		0.17	0	
40S ribosomal protein S3 (P23396)	12.4	6		1	3.7	
Electron transfer flavoprotein subunit alpha, mitochondrial (H0YK49)	0.30	0.08		0	0	
cDNA FLJ60124, highly similar to Mitochondrial dicarboxylate carrier (B4DLN1)	1.31	0.1		1.7	0	0.54
Leucine-rich PPR motif-containing protein 59, mitochondrial (Q96AG4)	1.3	0.3		0.13	0	0.04
Peroxiredoxin-5, mitochondrial (P30044)	0.33	0		0	0	

IAC of DmHsp22 and negative control with anti-Flag antibodies from 2 and 1 mg of S1300 g isolated from human HeLa cells are subjected to MS analysis. Proteins that were detected only in DmHsp22 expressing cells or that were detected with higher abundancy than the control were considered as specific interactors of DmHsp22. A total of 137 and 72 proteins were identified from two independent IACs and classified as mitochondrial partners of DmHsp22 using UniProt database bioinformatic resources. The highlighted proteins are the ATP synthase (complex V) subunits. Columns identified as DmHsp22-Flag and control show the emPAI index representing the abundance of detected proteins interacting with DmHsp22-Flag and control at Physiological condition. Heat shock +6 column shows the emPAI of detected proteins interacting with DmHsp22 following heat shock at 42°C for 1 hour and 6 hours recovery at 37°C.

MS analysis of the bound fraction from two independent experiments revealed a total of 951 and 404 different potential interacting-proteins for DmHsp22. Remarkably, 410 out of 951 and 157 out of 404 (42%), were RNPs and cytosolic proteins according to the Gene Ontology (GO) Cellular Component Information. Mitochondrial proteins were the second largest group of proteins detected, constituting 16% of total proteins. Cytoskeletal and Endoplasmic reticulum (ER) proteins followed with about 15 and 10% of the total proteins detected respectively. Other proteins from membranes, Golgi and secreted proteins accounted for about 17% of total detected proteins (Fig 2B). After removing unspecific interactions between mitochondrial partners of DmHsp22 compared to the control IAC, 139 out of 951 and 72 out of 404 proteins, respectively, were recognized as potential mitochondrial partners of DmHsp22 but with different efficiency (according to the abundancy of detected proteins through MS). Among all the detected proteins, 60 interacting-proteins of DmHsp22 were commonly detected in the two experiments. Abundancy of proteins interacting with DmHsp22 have been reported in Table 2 using the emPAI for estimation of absolute protein amount in proteomics analysis by the number of sequenced peptides per protein [44].

Distribution of detected proteins in two IACs in the different mitochondrial sub-compartments were identified using the UniProt identification system. 54% of detected proteins belonged to the inner mitochondrial membrane (IMM), 33.6% to the matrix and about 10.8% were associated with the outer mitochondrial membrane (OMM). Only a small amount, ~ 1.6%, were located in the intermembrane space (IMS) (Fig 2C). The most abundant detected proteins were ADP/ATP translocase 2 (SLC25A5) with 27.5 and 9.13 emPAI value, ADP/ATP translocase 3 (SLC25A6) with 16.2 and 7.26 emPAI value and Isoform 2 of ATPase family AAA domain-containing protein 3A with 12.4 and 4.8 emPAI value, even after considering the unspecific interactions with control beads. Interestingly, ATP synthase subunits formed the highest percentage of detected proteins in both experiments. These findings indicate that DmHsp22 is able to interact with a wide variety of mitochondrial proteins (Table 2).

### DmHsp22 interacts with proteins involved in different molecular functions

**Fig 3:**
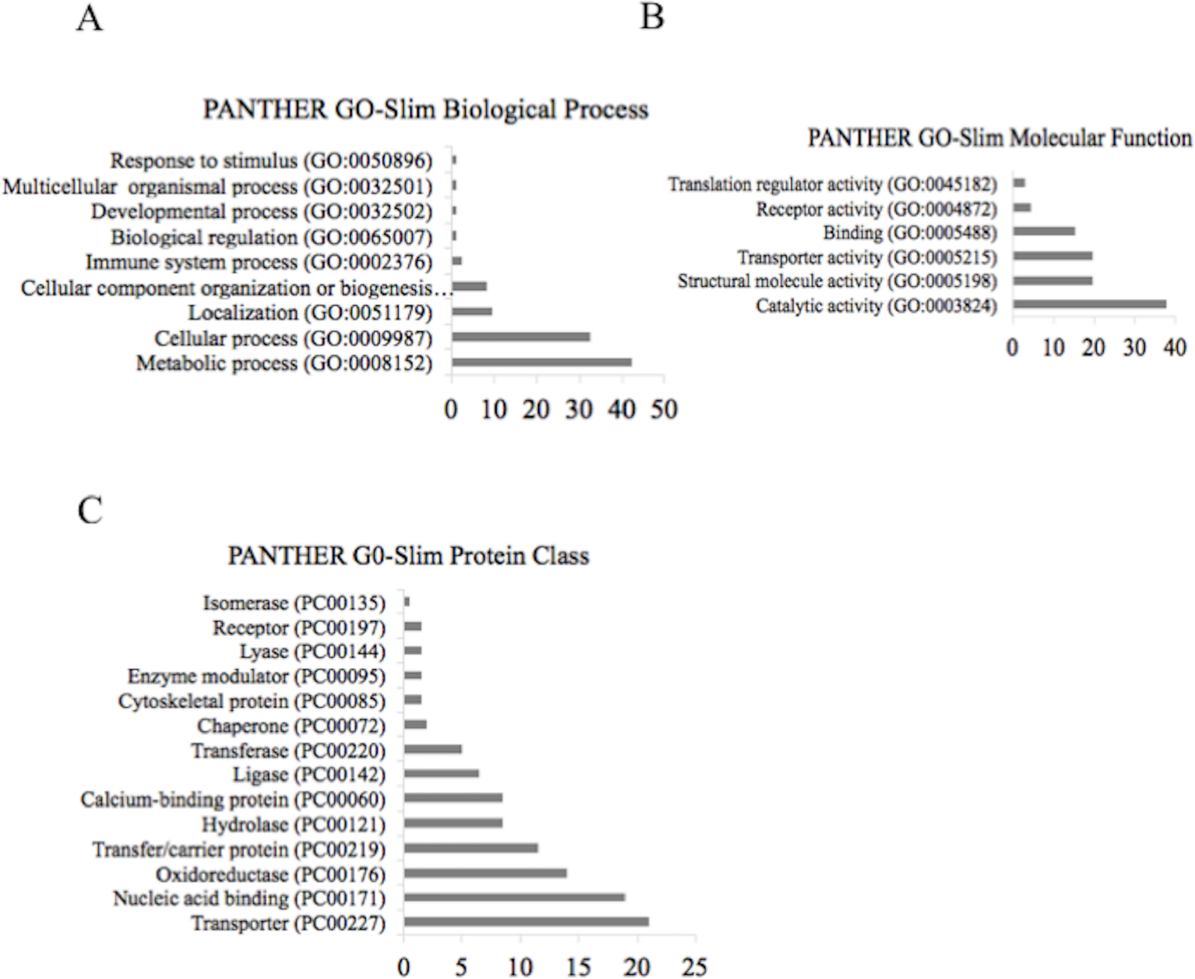
Gene Ontology analysis of DmHsp22-associated proteins. (A) The potential partners of DmHsp22 were regrouped in 3 categories according to their involvement in: biological processes, (B) molecular functions, (C) and protein classes. GO terms were used to describe the attributes of the DmHsp22 potential partners.

Proteins associated with DmHsp22 were automatically arranged based on their cellular and functional distribution using PANTHER Classification System (http://pantherdb.org) and according to information of the UniProt identifiers. The UniProt identification numbers of all mitochondrial DmHsp22-associated proteins were applied to the functional genomics resource tool GO (Gene Ontology). An over representation on “GO Biological Function” revealed involvement of detected proteins in many biological processes, with the most abundant proteins related to metabolic (GO:0008152) (42%) and cellular processes (GO:0009987) (33%). Other proteins associated with DmHsp22 were involved in different biological processes including localization (GO:0051179) (9.6%), cellular component organization or biogenesis (GO:0071840) (8.4%), immune system process (GO:0002376) (2.4%), response to stimulus (GO:0050896) (1.2%) and biological regulation (GO:0065007) (1.2%) (Fig 3A). Each category was divided into related subcategories shown in S1 Fig. For example, proteins classified in localization are divided in the protein localization (GO:0008104) and transporter (GO:0006810) subcategories. Proteins interacting with DmHsp22 were divided into six main groups according to their molecular functions. Among them about 38% were involved in catalytic activity (GO:0003824), while each of the structural (GO:0005198) and transporter proteins (GO:0005215) formed 20%. The remaining group was constituted of binding (GO:0005488), receptor (GO:0004872) and translational regulator activities (GO:0045182) with 15, 5 and 3% involvement, respectively (Fig 3B).

The resulting bar charts according to the PANTHER Classification System and UniProt information indicated that interacting proteins belong to the different protein classes and correlate to the various protein pathways. Interestingly, the largest category of protein classes with respect to their abundancy was associated to transporter proteins (PC00227), nucleic acid binding (PC00171) and oxidoreductase protein class (PC00176) with 21, 19 and 14%, respectively. Other protein classes are presented in Fig 3C.

According to the PANTHER Protein Pathways, ATP synthase pathway (P02721) is the most abundant pathway (21.1%) in which mitochondrial DmHsp22-associated proteins are involved. TCA cycle (P00051) and Huntington disease (P00029) each with 10.5% are among the second highly abundant pathways in which DmHsp22-associated proteins are involved. Moreover, different types and isoforms of actin, tubulin and myosin formed a significant part of DmHsp22’s partners, although they are not mitochondrial members. These cytoplasmic structural proteins seem to be involved in mitochondrial trafficking and may be crucial to position mitochondria with specific subcellular compartments.

### ATP synthase subunits are the main DmHsp22-binding proteins

**Fig 4:**
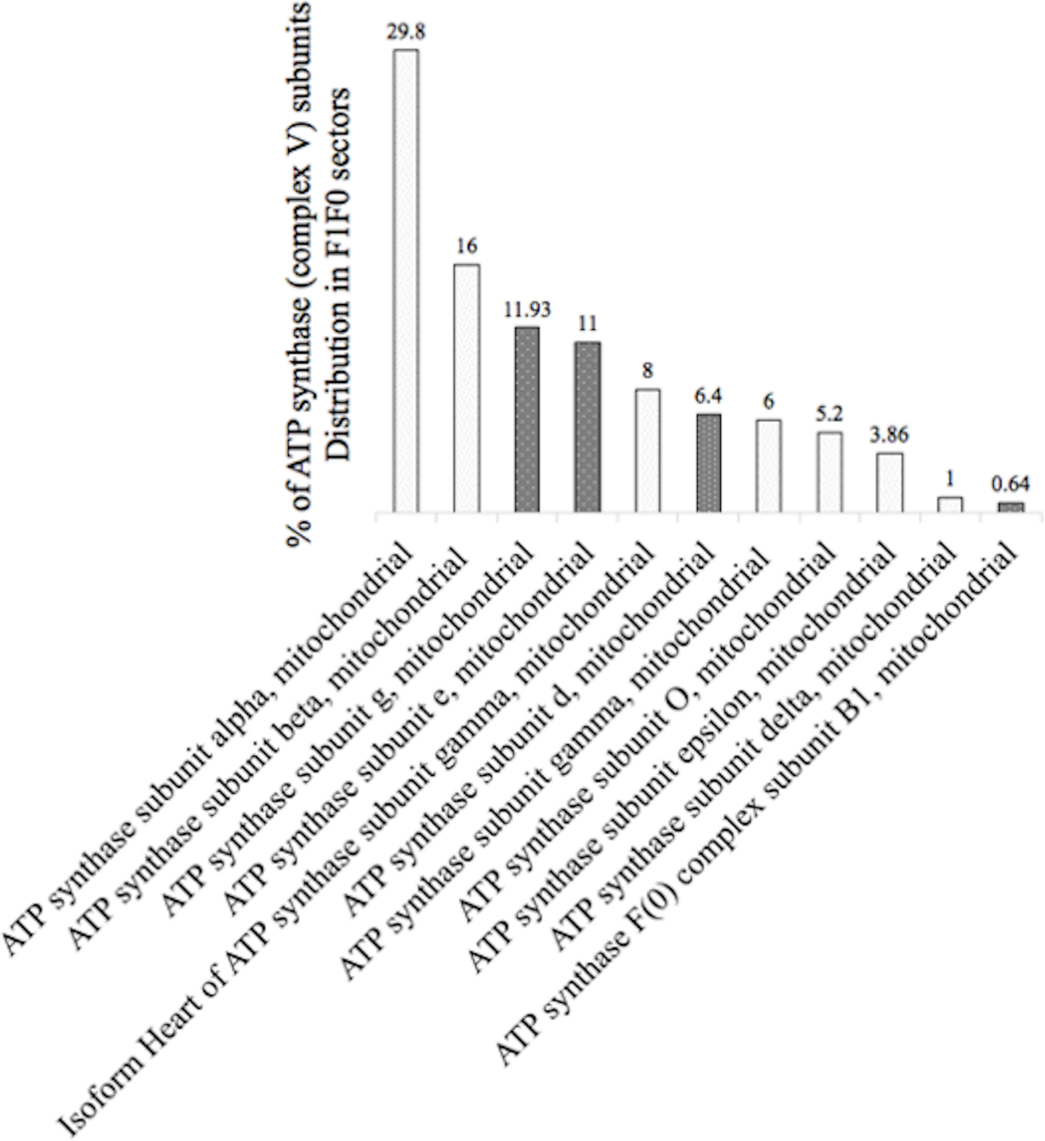
Abundancy of ATP synthase (Complex V) subunits and subunit composition of DmHsp22-associated partners. Detected subunits of the ATP synthase machinery as mitochondrial partners of DmHsp22 have been summarized in the bar chart from the highest to the lowest percentage of detected proteins. Subunits related to the F1 and F0 sub-complexes have been identified in white and black background bar charts according to their association to the F1 or F0 sub-complexes respectively.

The mitochondrial ATP synthase machinery (Complex V) consists of two well defined sub-complexes entities F1 and F0. F1 is a soluble protein localized in the mitochondrial matrix and comprises 6 different subunits including alpha, beta, gamma, delta, epsilon and OSCP, while F0 bound to the IMM and consists of a, b, d, F6, IF_1_, e, g, f and A6L [46, 47]. The Mass Spectrometry analysis of the two independent IACs detected 11 subunits of ATP synthase machinery that represent 7.9 and 9.7% of the total mitochondrial proteins interacting with DmHsp22 and are mostly localized in the F1 sub-complex. ATP synthase subunits alpha and beta were the most abundant partners detected, representing 29.8 and 16% of total ATP synthase subunits recovered, respectively (Fig 4). Specifically, ATP synthase subunit alpha with emPAI factor (protein abundancy in MS result) of 7.2 in one IAC and 21.5 in the other, was the most abundant detected partners among ATP synthase subunits (Table 2). Subunit beta with emPAI factor 10.6 in one IAC and 10 in the other, was the second most abundant of ATP synthase subunits detected. ATP synthase subunits g and e each formed about 11% of complex V partners. Specifically, abundancy of subunit g was 10.2 and represented the third most abundant group of ATP synthase subunits interacting with DmHsp22. Subunit gamma of ATP synthase was detected with emPAI factor 5.5 and 2.5. The heart isoform of ATP synthase subunit gamma was detected with emPAI index 4.8 and 2.5 from two different mass spectrometry analyses. Mitochondrial ATP synthase subunit e was detected with 4.51 and 8.35 abundancies. Mitochondrial ATP synthase subunit d and ATP synthase subunit epsilon were detected with 3.8 and 3.65 abundancy at the first IAC, and only subunit d with emPAI index 3.5 was detected in the second IAC. ATP synthase subunit O was detected with emPAI indexes 2.12 and 3.6, respectively (Fig 4). All the ATP synthase subunits detected as DmHsp22-binding proteins have been highlighted in the Table 2.

### Heat shock influences stability of mitochondrial partners of DmHsp22

**Fig 5:**
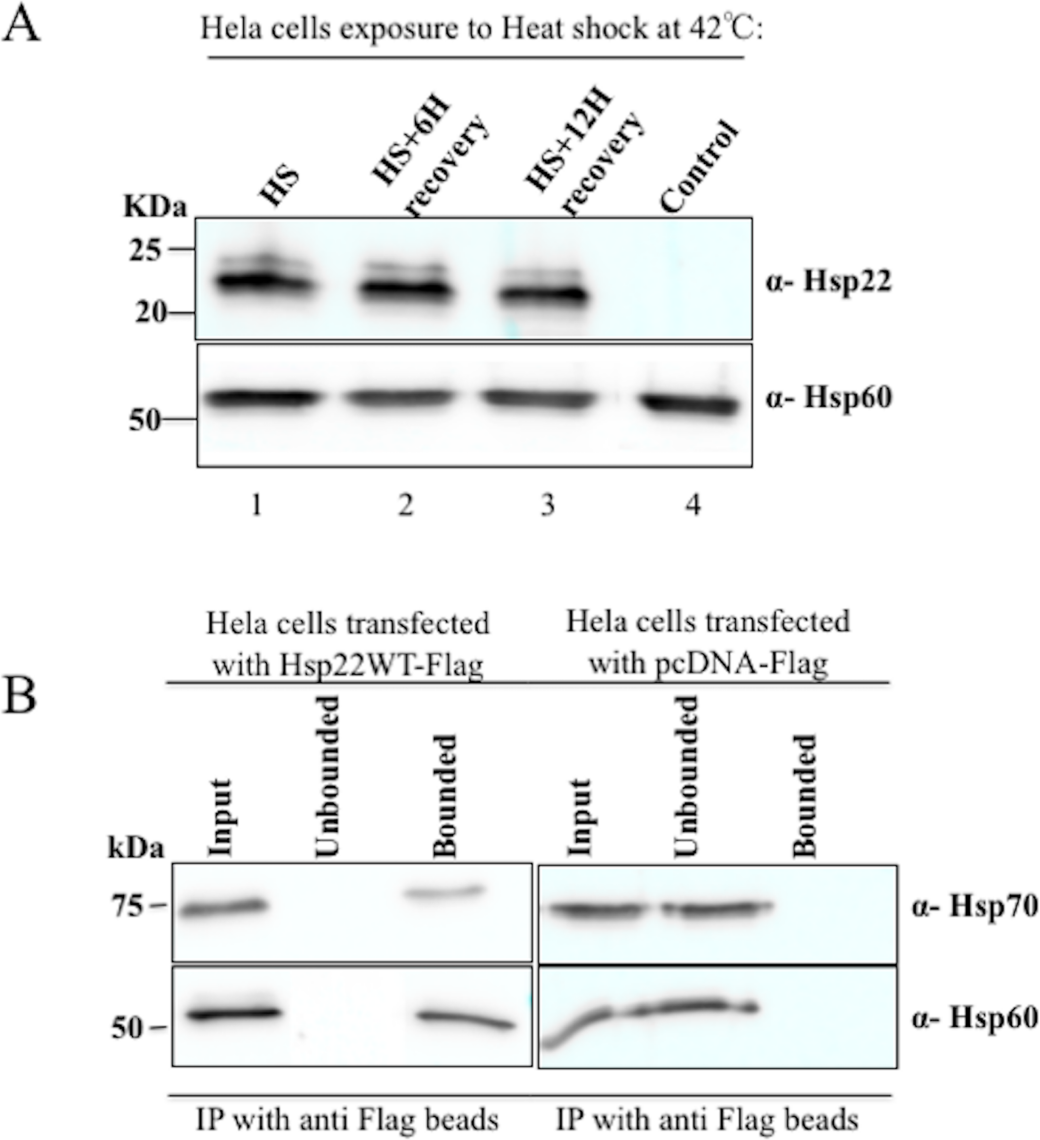
Heat shock and recovery does not influence expression levels of DmHsp22. Confirmation of DmHsp22’s interaction with Hsp60 and Hsp70. (A) HeLa cells expressing DmHsp22 and pcDNA vector were harvested and analyzed for DmHsp22 expression at 0, 6 and 12 hours post-recovery after heat shock by western blot. HS (1 hour heat shock without any recovery), HS+6H recovery (1 hour heat shock at 42°C and 6 hours recovery at 37°C), HS +12H recovery (1 hour heat shock at 42°C and 12 hours recovery at 37°C). anti-Hsp60 was used as loading control. (B) IAC on S1300 g of HeLa cells expressing DmHsp22 as well as HeLa cells expressing control vector under heat shock (HS) conditions, where cells were treated at 42°C for 1 hour followed by 6 hours post-recovery at 37°C and detecting Hsp70 and Hsp60. Results are representative of 4 separate experiments (N = 4).

In order to see if heat shock (HS) might modify the stability of DmHsp22 partners, HeLa cells were exposed to a 42°C heat shock during 1 hour. Immuno-detection revealed that the expression levels of DmHsp22 are not impacted by HS, even after 6 and 12h recovery (Fig 5A). To identify which protein partners stably interact with DmHsp22 after stress, IAC coupled to MS was performed after HS and 6 h recovery. More than 50% of the potential partners were stably bound to the sHsp following the thermal stress. However, the abundancy or content (emPAI factor) of detected proteins was reduced after heat shock and recovery for 6 hours, which suggests that potential partners of DmHsp22 are degraded under stress condition (Table 2). ATP synthase subunits form 18.75% of the detected proteins after HS and 6 h recovery. The mitochondrial 60 kDa heat shock protein, ATP synthase subunit alpha and elongation factor Tu were the most abundant proteins bound to DmHsp22 after HS and after 6h-recovery. To confirm interaction of Hsp60 and Hsp70 (which detected with high abundancy and are member of chaperone family) with DmHsp22, IAC experiment was performed on the S1300 g (Fig 5B).

ATP synthase subunits beta and g both with emPAI index about 6 were among the proteins that bound to DmHsp22 with high tendency after 6 hours of recovery post HS (Table 2). The remaining proteins displaying high efficiency were the mitochondrial ATP synthase subunit e (emPAI index 6), mitochondrial ATP synthase subunit O (with emPAI index 3.16 for Hsp22-Flag and 0.65 for control beads), as well as ADP/ATP translocase2 and ADP/ATP translocase 3 (abundancy of each was 3 and 2.4 after considering abundancy of unspecific interactions, respectively). The characteristics of proteins that stably interacted with DmHsp22 was achieved by analysis of the 48 detected proteins using the PANTHER Classification System. These proteins were mostly localized in the IMM and involved in molecular functions including catalytic activity (18), transports (11), translation regulator activity (2), structural molecules (8), receptors (3) and binding activity (6) (S1A and S1D Fig). According to their involvement in biological processes, they are mostly involved in metabolic process (GO:0008152) and based on their protein classification, they are mostly transporter proteins (PC00227) (S1B and S1C Fig).

### DmHsp22 effects on mitochondrial oxygen consumption and ATP content of cells

**Fig 6:**
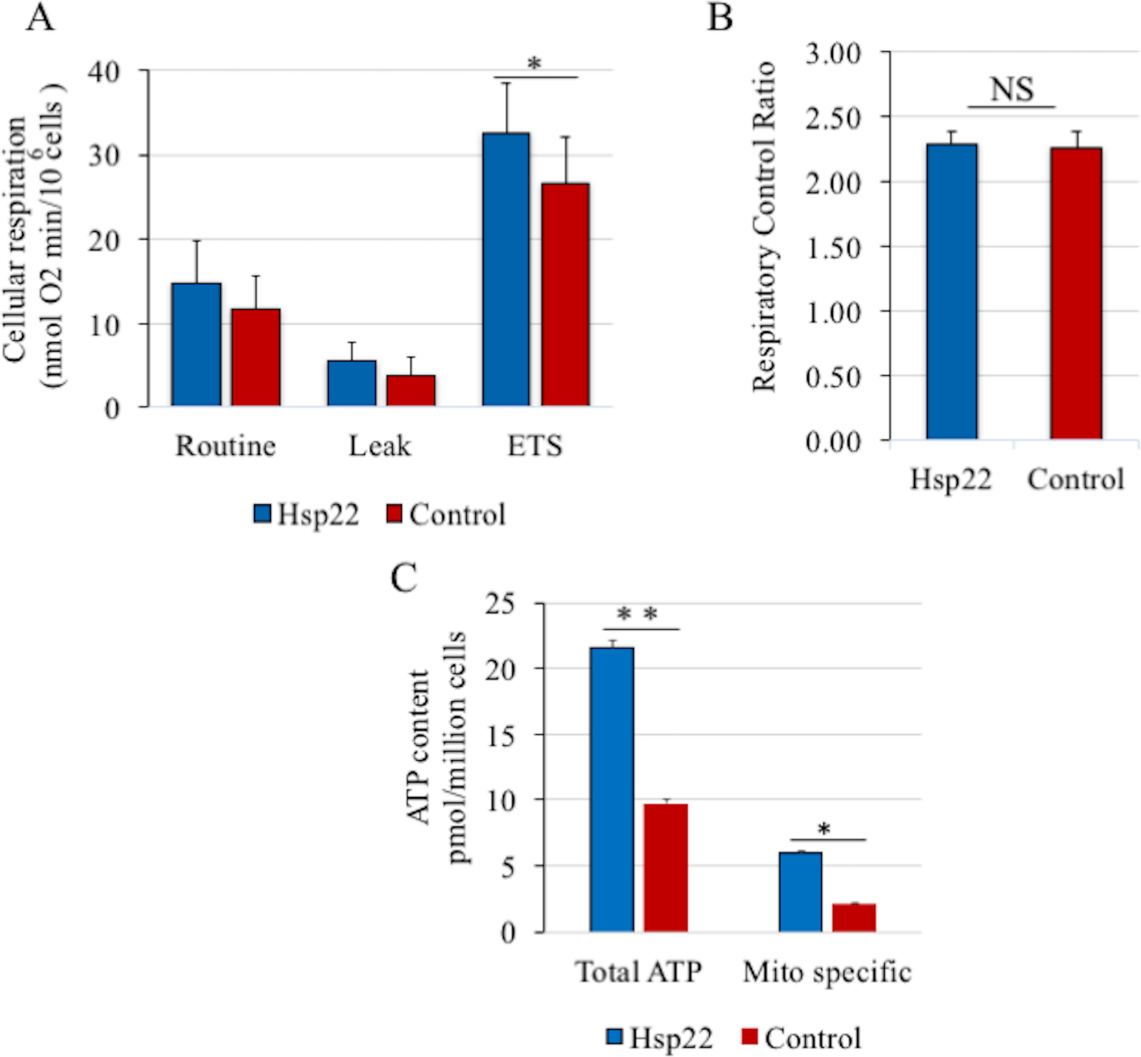
Mitochondrial oxygen consumption in intact cells and cellular ATP concentration. (A) ROUTINE, LEAK and ETS respiration rates measured in HeLa cells transfected with DmHsp22 (blue) as compared to the cells transfected with empty vector (red). Only ETS respiration was significantly higher in transfected cells (N = 7). (B) The respiratory control ratio (RCR = ETS/LEAK) is not different between HeLa cells transfected with DmHsp22 compared to the control cells (N = 7). (C) Cellular ATP concentration due to mitochondrial phosphorylation of ADP into ATP is higher in cells transfected with DmHsp22 as compared to the control cells (N = 4). Data are presented as means ± SEM. P*< 0.05; P** < 0.01, according to t-test.

Given that several subunits of ATP synthase protein were found to interact with DmHsp22, we decided to measure mitochondrial oxygen consumption in cells expressing the small Hsp. Sequential injections of oligomycin, rotenone and antimycin A as well as FCCP were performed. Different steps of mitochondrial respiration rates i.e. ROUTINE (oxygen consumption supported by endogenous substrates or glucose in the culture media), LEAK (oxygen consumption maintained to compensate the proton leak during non-phopshorylating respiration) and ETS (non-coupled respiration representing maximal oxygen consumption capacity of the ETS) were measured, and only the ETS respiration (non-coupled) was significantly increased in DmHsp22 expressing cells as compared to the control cells (Fig 6A). However, expression of DmHsp22 did not change the respiration control ratio (RCR), suggesting that this mitochondrial heat shock protein does not alter the mitochondrial membrane potential or other indicator of the mitochondrial activity (Fig 6B).

Finally, ATP content of cells was measured in the cells transfected with DmHsp22. Interestingly, both total ATP levels and the specific mitochondrial ATP levels were affected and significantly increased in DmHsp22 transfected cells (Fig 6C).

### DmHsp22 is an efficient chaperone in human cells

**Fig 7:**
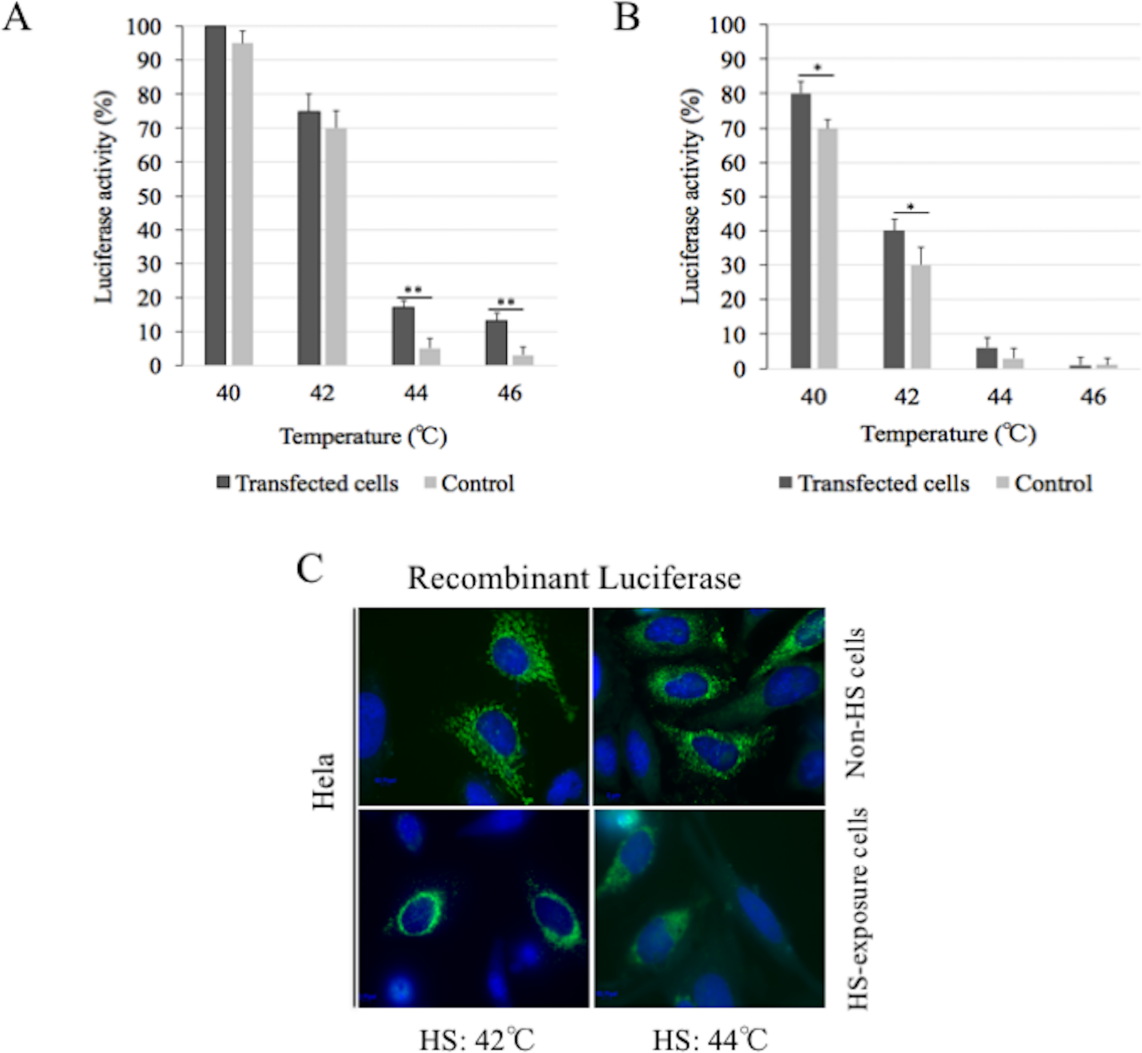
DmHsp22 is an important factor in thermotolerance under heat stress condition. **(A)** The DmHsp22 (black) and control vector (gray) transfected cells in cells transiently expressing mitochondrial luciferase were cultured and transfected as described in Materials and Methods. Cells were exposed to 40, 42, 44 or 46°C for 30 and (B) 60 minutes in presence of 20 μg. mL^-1^ cycloheximide. Following heat exposure, cells recovered for 6 hours at 37°C, were lysed and assayed for luciferase activity. Data are presented as means and SD of four replicates. P*< 0.05; P** < 0.01 and P***<0.001 according to two-way ANOVA (A-B). (C) Fluorescence microscopy of HeLa cells expressing recombinant luciferase. Top 2 rows represent Non-HS cells and bottom 2 rows represent HS cells at 44°C for 30 and 60 min.

To experimentally test the chaperone-like activity of DmHsp22 in human cells, its ability to reactivate mitochondrial targeting luciferase was assessed in HeLa cells transiently expressing DmHsp22. Heat shock on HeLa cells as low as 40°C for 30 min and 1 hour was sufficient to inactivate part of luciferase activity. Indeed, luciferase lost 5% and 30% of its initial activity after 30 and 60 min at 40°C, respectively and 30% and 70% of its initial activity after 30 min (Fig 7A) and 60 min (Fig 7B) of heat treatment at 42°C, respectively. Luciferase activity was higher in HeLa cells expressing DmHsp22 compared to control cells under heat stress conditions. Almost no luciferase activity was observed in HeLa cells after 30 and 60 min at 44 and 46°C, respectively (Fig 7A and 7B) (5% or less). Cells expressing DmHsp22 were less influenced by the heat stress (40–46°C) in the period of inactivation compared to the control cells. Luciferase regained 5% of its initial activity in presence of DmHsp22 after 30 min denaturation at both 40 and 42°C, and 10% after 60 min at both temperatures. DmHsp22 was also more effective after treatment for 30 min at 44 and 46°C recovering 12 and 10% of luciferase activity, respectively, compared to 5% or less for controls. These results show that DmHsp22 can protect substrates such as luciferase from inactivation at temperatures as high as 46°C. Confocal analysis revealed the mitochondrial distribution of mitochondrial luciferase after heat shock exposure (Fig 7C).

## Discussion

Mitochondria in addition to be the cellular powerhouse are involved in several important biological functions [21, 22, 48, 49]. Disruption of mitochondrial functions is a significant triggering factor for many diseases including cancer [50, 51]. The mitochondrial proteome changes under different pathological conditions as well as during aging. For example, The level of ATP synthase subunits has been reported to vary in response to aging [52–54]. The *Drosophila melanogaster* small heat shock protein Hsp22 is localized in mitochondria [38] and its expression has been shown to be induced in response to various stresses such as heat, oxidative stress and during aging [55–59]. Interestingly, flies over-expressing DmHsp22 have an increased longevity and show a faster response to the stress compared to normal flies [55, 60]. Our approach in choosing a heterologous system is evidenced by the previous studies of Wadhwa et. al (2010) who confirmed cross-species effects of DmHsp22’s expression in HeLa cells as well as in Drosophila. DmHsp22 is functionally active in human fibroblasts at the temperature of growth and extends lifespan and slows down the aging process at 37°C, as shown by lower levels of senescence markers [61]. Following expression in mammalian cells, DmHsp22 has been found in the mitochondria as well as in the soluble fraction. We therefore used the S1300 supernatant to get a first look at all the possible interactors. 139 and 72 DmHsp22-associated proteins with various functional classifications were identified by transiently expressing it in a mammalian cell line (HeLa cells). In a study on the bacteria *Synechocystis*, Basha et al. [62] identified 42 proteins interacting with the unique sHsp Hsp16.6. These proteins were involved in a great variety of cellular activities [62].

Most of the proteins interacting with DmHsp22 are transporters localized in IMM and components of ATP synthase and TCA cycle. This is consistent with our recent observation that flies over-expressing DmHsp22 show different expression levels of TCA cycle and ETS proteins [18]. In another study performed on expression of DmHsp22 in oenocytes, accumulation of age-pigment and superoxide was decreased, which suggests involvement of Hsp22 in reducing mitochondrial metabolism and ROS production [63]. Recently, chaperone activity of DmHsp22 against different protein substrates have been reported *in vitro* [64]. We suggest here that the interaction of DmHsp22 with ATP synthase subunits may influence the assembly of the ETS due to its chaperone activity. Among the DmHsp22 interacting proteins, subunits alpha, beta and gamma were the most abundant proteins detected as members of mitochondrial complex V. Interestingly, there were no differences between proteins interacting with DmHsp22 during heat shock or under normal conditions. The influence of DmHsp22 on mitochondrial oxygen consumption and ATP content is also of interest. DmHsp22 significantly increases mitochondrial non-coupled respiration (with FCCP), which represents the maximum oxidative capacity of the electron transport system. Moreover, mitochondrial ATP levels increased after expression of DmHsp22. ATP synthase subunits are affected by age-dependent processes across all species, especially the beta and alpha subunits [65–69]. This suggests that DmHsp22 could be involved in the modulation of ATP synthase during aging. Indeed, ATP synthase is among the main mitochondrial age-related targets of post-translational modifications, resulting in loss of ATP level in aged human cells [70–72]. This is consistent with the role of DmHsp22 in the aging process through its interaction with members of the ATP synthase machinery. Both F0 and F1 subunits of complex V have been detected among the potential partners of DmHsp22. Although DmHsp22 improves maximal mitochondrial oxygen consumption capacity and ATP content, RCR was not influenced by DmHsp22 expression. This suggests that DmHsp22 may be involved in assembly of complex V since the content of detected proteins are lower during heat shock conditions and recovery.

Over-expressing DmHsp22 in flies had significant effects on mitochondrial transcripts, especially on those involved in the ETS, and the TCA cycle, as well as on mitochondrial Hsp70 (mtHsp70) [59, 63, 73–77]. Proteins from the ETS, the TCA cycle and the mitochondrial Hsp70 were among the proteins up-regulated in flies over-expressing DmHsp22, as seen in IEF/SDS gel analysis [18]. Interestingly, some of the DmHsp22-associated proteins noted in the IACs were similar to the proteins up-regulated in the flies over-expressing DmHsp22 (DmHsp22^+^). Among them were mitochondrial Hsp70 (HspA9/CG8542), ATP synthase subunit alpha (ATP5A1)/CG3612, ATP synthase subunit ß (ATP5B/CG11154), glutamate dehydrogenase (GLUD1)/CG5320, mitochondrial elongation factor Tu (TUFM)/CG6050, and different types of cytoskeletal proteins including tubulin, myosin and ferritin.

ADP/ATP translocase 2 (SLC25A5) (P05141) and ADP/ATP translocase 3 (SLC25A6) (P12236) were among the most abundant proteins detected in two independent MS analysis. They were located at the inner mitochondrial membrane and are characterized as mitochondrial carrier [78]. Their detection with the high abundancy is intriguing, especially because they are members of solute carrier family 25 (SLC25) responsible for shuttling a large variety of molecules across the inner mitochondrial membrane [79–81]. SLC25A5 (solute carrier family 25, member 5) is highly expressed in cancer cells [82], and SLC25A6 (solute carrier family 25, member 6) is an enzyme involved in exchanging ADP/ATP through mitochondrial membranes [82, 83] and its expression has been shown to induce apoptosis in cellular culture [84]. Thus, there is likely an involvement of DmHsp22 in exchanging metabolites as well as ADP and ATP between mitochondria and cytoplasm. Further investigations and experiments are necessary to validate the exact role of DmHsp22 regarding its interaction with the ADP/ATP translocases 2 and 3.

DmHsp22, DmHsp60 and mtDmHsp70 have been shown to be up-regulated following stress in flies [74, 76]. Interestingly, mtHsp70 mRNA was the second highest spot detected in DmHsp22^+^ flies [8, 75, 76, 85]. It should be mentioned that ATP synthase subunit alpha (TP5A1/CG3612) and ATP synthase ß subunit (ATP5B/CG11154) were up-regulated with high intensity (28.5±7.3 and 16.4 ±4.8) in flies over- expressing DmHsp22 respectively compared to the control [18]. The involvement of DmHsp22 in the mtUPR was suggested together with Hsp60 and mtHsp70 [59, 63, 74]. In our IAC experiments, chaperone proteins Hsp60 and mtHsp70 displayed high efficiency even during heat shock condition as mitochondrial partners of DmHsp22. This suggests their involvement in the re-folding processes together with their chaperone-like activity, as they are ATP-dependent chaperones. To our knowledge, this is the first time that the effect of DmHsp22 over-expression on respiration and ATP content has been reported. DmHsp22 may interact with subunits to help with their assembly and may protect them during stress.

## Supporting information

S1 Fig Biological relevance of DmHsP22-associated proteins. (A) DmHsp22 associated proteins have been categorized into 9 major groups and 18 sub-groups of biological processes using PANTHER classification system. (B) The associated proteins have also been categorized into 6 main groups and 14 sub-groups of molecular functions using PANTHER classification system.
(TIFF)

S2 Fig Mitochondrial distribution and biological relevance of DmHsp22-associated proteins following heat shock exposure and recovery. (A) Mitochondrial distribution of DmHsp22-associated proteins following HS for 1 hour at 42°C and 6 hours recovery in different sub-compartments of mitochondria were identified using information of UniProt. The partners of DmHsp22 were regrouped in 3 categories according to their involvement in: (B) protein classes, (C) biological process, and (D) molecular functions. GO terms were used to describe the attributes of the DmHsp22 partners.
(TIFF)
