# Supplementary figures and images for: Identification of proteins interacting with the mitochondrial small heat shock protein Hsp22 of Drosophila melanogaster: Implication in mitochondrial homeostasis
Source: PLoS One. 2018 Mar 6;13(3):e0193771. doi: 10.1371/journal.pone.0193771 (PMC5839585; doi:10.1371/journal.pone.0193771)

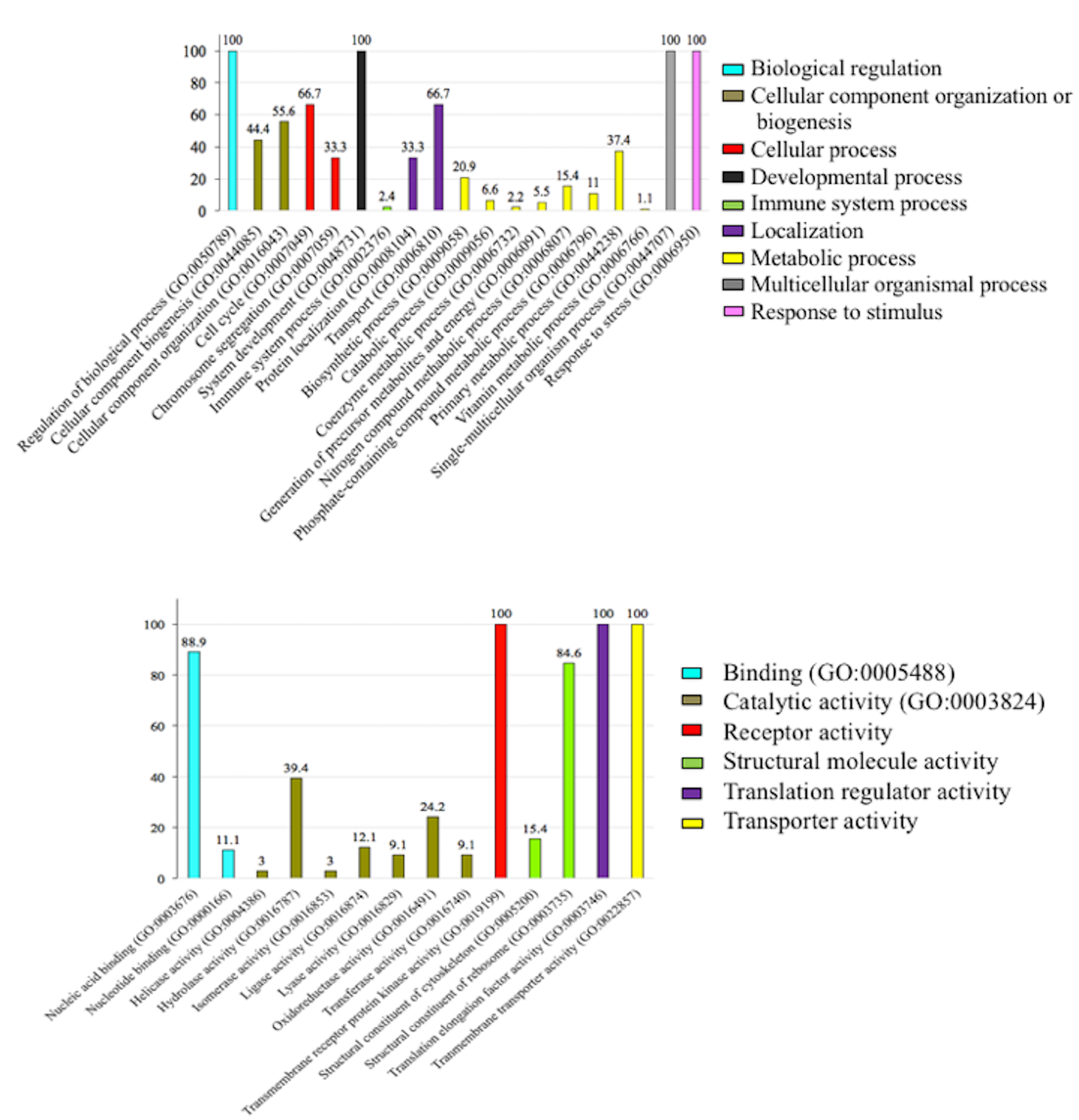

Supplement: S1 Fig — (A) DmHsp22 associated proteins have been categorized into 9 major groups and 18 sub-groups of biological processes using PANTHER classification system. (B) The associated proteins have also been categorized into 6 main groups and 14 sub-groups of molecular functions using PANTHER classification system. (TIFF) [file pone.0193771.s001.tiff]

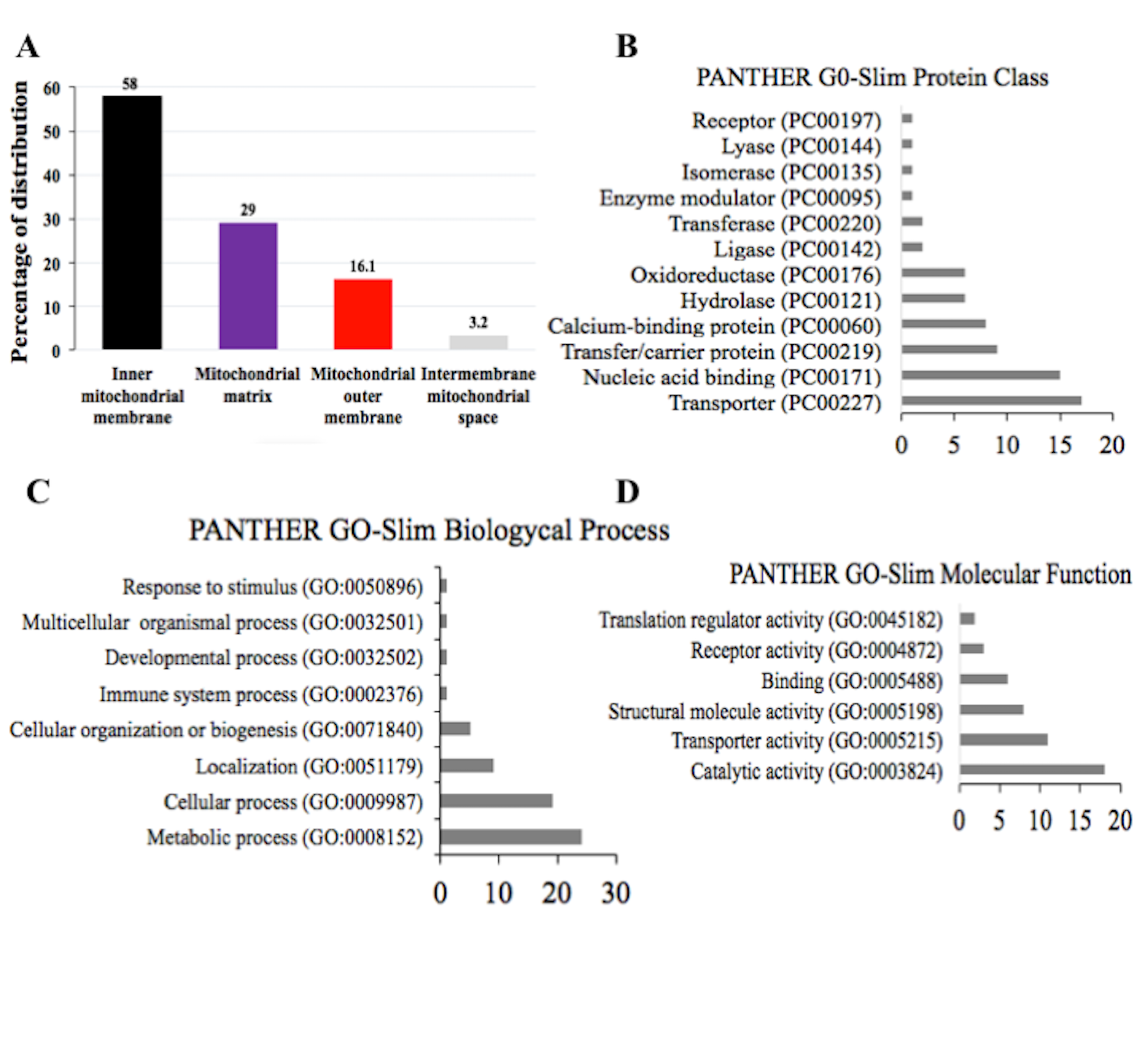

Supplement: S2 Fig — (A) Mitochondrial distribution of DmHsp22-associated proteins following HS for 1 hour at 42°C and 6 hours recovery in different sub-compartments of mitochondria were identified using information of UniProt. The partners of DmHsp22 were regrouped in 3 categories according to their involvement in: (B) protein classes, (C) biological process, and (D) molecular functions. GO terms were used to describe the attributes of the DmHsp22 partners. (TIFF) [file pone.0193771.s002.tiff]
